# Supplementary material for: A Computational Approach to Identification of Candidate Biomarkers in High-Dimensional Molecular Data
Source: Diagnostics (Basel). 2022 Aug 18;12(8):1997. doi: 10.3390/diagnostics12081997 (PMC9407361; doi:10.3390/diagnostics12081997)
Supplement: Supplementary file 1 [file diagnostics-12-01997-s001.zip › diagnostics-1827412-supplementary/Supplementary Table Legends.pdf]

Supplementary Table Legends

Supplementary Table S1. List of immune-function genes. A table of the extracted immune-function genes used for feature selection and differential expression analysis. The first column provides a list of all 923 immune-function genes extracted from the TCGA PRAD samples. The second column provides a list of the remaining genes after filtering for low expression.

Supplementary Table S2. System requirements for *MFeaST*. Minimum and recommended specifications and requirements for running *MFeaST* on Mac or Windows.

Supplementary Table S3. Feature selection results. *MFeaST* ranking results for each gene in A) Luminal A vs Basal, B) Luminal B vs Basal, and C) Luminal A vs Luminal B comparisons. Rows represent ranked genes and columns represent the overall rank and median expression levels of that gene for each comparison group (A-C). Genes that contain an asterisk (*) were handpicked through *MFeaST* visualization analysis and were used for building the classification models.

Supplementary Table S4. Differential expression analysis results. Results of differential expression analysis performed using the edgeR package (version 3.25.8) are presented in order of ascending FDR value. Abbreviations: $\log_2$ fold change (logFC), false discovery rate (FDR).

Supplementary Table S5. Feature selection and differential expression results comparison. Features selected with *MFeaST* are compared to the differentially expressed genes for comparisons: A) Luminal A vs Basal, B) Luminal B vs Basal, and C)

Luminal A vs Luminal B. Rows represent a gene and columns represent if for each comparison (A-C) the gene appeared 1) only in the feature selected set, 2) both feature selected and differentially expressed set (intersection), or 3) only in the differentially expressed set.
