# Supplementary material for: A Computational Approach to Identification of Candidate Biomarkers in High-Dimensional Molecular Data
Source: Diagnostics (Basel). 2022 Aug 18;12(8):1997. doi: 10.3390/diagnostics12081997 (PMC9407361; doi:10.3390/diagnostics12081997)
Supplement: Supplementary file 1 [file diagnostics-12-01997-s001.zip › diagnostics-1827412-supplementary/Table S2. System requirements for MFeaST.pdf]

Table S2. System requirements for MFeaST

Specification	Minimum requirement	Recommended
Operating System	Windows 7 SP 1 or macOS 10.14	Windows 10 or MacOS 11
Disk space	10 Gb	15 Gb
RAM	4 Gb	8 Gb
CPU	Any Intel or AMD x86-64 processor	Any Intel or AMD x86-64 processor with four logical cores and AVX2 instruction set support
